# Rare presentation of dandy-walker variant syndrome associated with leigh syndrome: a promising therapeutic approach for prognosis in children related in a case report

**DOI:** 10.1093/omcr/omaf061

**Published:** 2025-06-27

**Authors:** Bianca Frigo Pires, Graziele Ines Silva Lopo, Anaí Ramoa Siqueira, Luiza Resende Felisberto, Rafaella Simão Martini, Ana Beatriz Valim Alves, Amanda Carolina Rodrigues Silva, Flávia Xavier Lima Carvalho, Aline Pirola Aliseda, Mariane Martinhon Martins, Carla Kreuzberg Salgado, Salum Bueno da Silveira Junior, Camila Garcia Ferrari Jacob

**Affiliations:** Department of pediatrics, Hospital das Clinicas de Marília, R. Orlando Riguetti, – São Paulo, Marília 227, Brazil; Department of pediatrics, Hospital das Clinicas de Marília, R. Orlando Riguetti, – São Paulo, Marília 227, Brazil; Department of pediatrics, Hospital das Clinicas de Marília, R. Orlando Riguetti, – São Paulo, Marília 227, Brazil; Department of pediatrics, Hospital das Clinicas de Marília, R. Orlando Riguetti, – São Paulo, Marília 227, Brazil; Department of pediatrics, Hospital das Clinicas de Marília, R. Orlando Riguetti, – São Paulo, Marília 227, Brazil; Department of pediatrics, Hospital das Clinicas de Marília, R. Orlando Riguetti, – São Paulo, Marília 227, Brazil; Department of pediatrics, Hospital das Clinicas de Marília, R. Orlando Riguetti, – São Paulo, Marília 227, Brazil; Department of pediatrics, Hospital das Clinicas de Marília, R. Orlando Riguetti, – São Paulo, Marília 227, Brazil; Department of pediatrics, Hospital das Clinicas de Marília, R. Orlando Riguetti, – São Paulo, Marília 227, Brazil; Department of pediatrics, Hospital das Clinicas de Marília, R. Orlando Riguetti, – São Paulo, Marília 227, Brazil; Department of pediatrics, Hospital das Clinicas de Marília, R. Orlando Riguetti, – São Paulo, Marília 227, Brazil; Department of pediatrics, Hospital das Clinicas de Marília, R. Orlando Riguetti, – São Paulo, Marília 227, Brazil; Department of pediatrics, Hospital das Clinicas de Marília, R. Orlando Riguetti, – São Paulo, Marília 227, Brazil

**Keywords:** dandy-walker variant, leigh syndrome, pediatric syndrome

## Abstract

Introduction: Dandy-Walker syndrome (D-WS) is a rare congenital brain anomaly that primarily impacts the fourth ventricle and cerebellum. Its variant is even rarer and includes cerebellar dysgenesis, with possible posterior fossa enlargement and variable cerebellar vermis hypoplasia. Case report: We present the case of a patient diagnosed late with the Dandy-Walker Syndrome variant, associated with Leigh Syndrome, at a tertiary hospital. The patient received an optimized, multidisciplinary treatment approach to improve prognosis. Discussion: Early intervention in pediatric neurodegenerative diseases through a multidisciplinary team that includes medical, speech therapy, and physiotherapy support is crucial for a better prognosis in these cases.

## Introduction

Dandy-Walker syndrome (D-WS) is a congenital brain condition affecting the cerebellum and fourth ventricle. Its variant, which is exceptionally rare, often features cerebellar dysgenesis along with potential posterior fossa enlargement and variable degrees of vermis hypoplasia. This variant frequently coexists with over 50 genetic syndromes and is commonly associated with karyotype 3 abnormalities [1]. When diagnosed early, particularly in families with a history of developmental and structural anomalies, the likelihood of identifying affected individuals increases significantly.

The objective of this article is to describe the case of a rare disease in pediatric patients and emphasize the need for early investigation, as well as its multidisciplinary management to improve prognosis.

## Case report

A 9-year-old girl arrived at the pediatric emergency department presenting with dyspnea and agitation. Her mother reported that similar symptoms had appeared four days earlier and were treated with azithromycin, salbutamol, and inhalation therapy without improvement. On examination, the child, who had a tracheostomy, appeared hydrated, active, and dyspneic. Cardiac auscultation was unremarkable, and lung examination revealed rhonchi and wheezing. Treatment with salbutamol, continuous saline nebulization, and a chest X-ray were administered. Laboratory tests showed a CRP level of 15.6, with other parameters within normal limits.

The mother noted that the child exhibited normal neuropsychomotor development (NPMD) until age three, when she showed slight speech delays resembling those of her late sister, who died from complications of Dandy-Walker Syndrome. Neurological examination revealed slowed speech, bilateral hyporeflexia in the lower limbs, and an unsteady gait. Initial brain imaging and EEG results were unremarkable. Two years later, the patient presented with reduced consciousness and hypoxemia, necessitating intubation and admission to the ICU for two months. The clinical history and family background prompted an investigation into Dandy-Walker Syndrome, leading to a confirmed diagnosis along with Leigh Syndrome.

**Figure 1:**
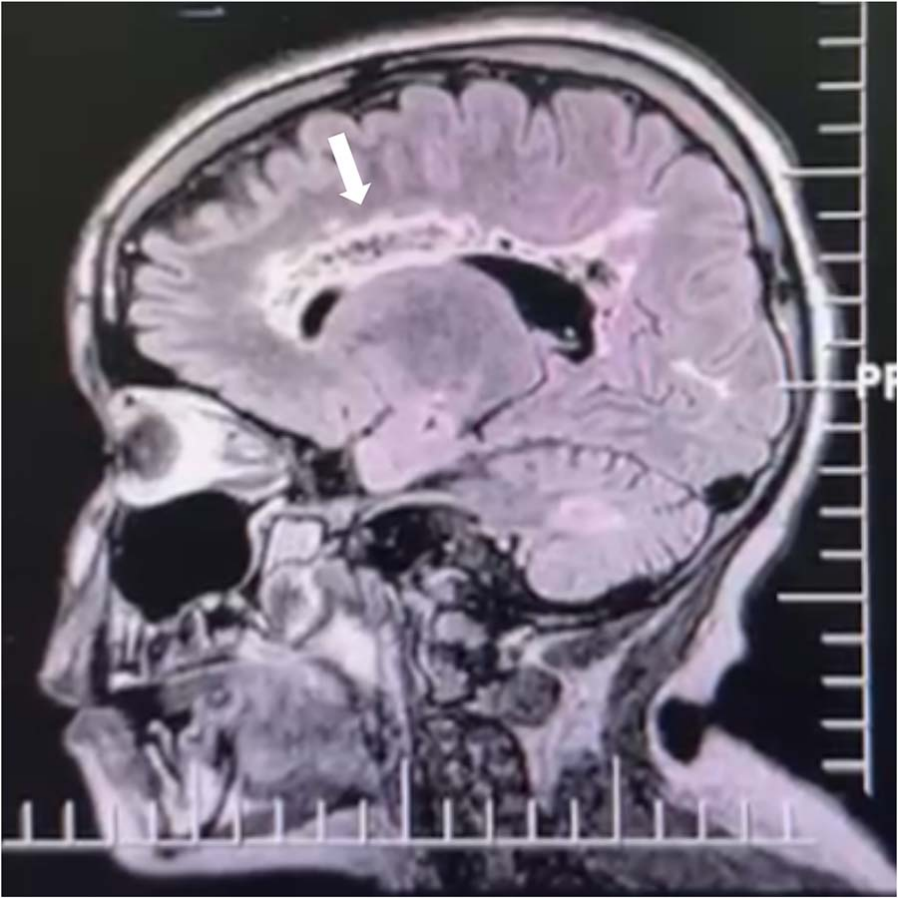
Magnetic resonance imaging (MRI) of the skull shows lesions with hyperintense signal, affecting the periventricular white matter of the corpus callosum up to the occipital lobe, sparing the temporal lobes and posterior fossa symmetrically.

During her hospitalization, continuous medications were maintained, and arginine and creatine were introduced. Multidisciplinary therapy was also intensified. A CT scan showed hypoattenuating areas in the white matter of the semioval centers, likely indicating gliosis. Figure 1.

Despite the progressive nature of the syndrome, the patient improved cognitively and regained consciousness, though she continues to experience motor difficulties and cerebellar ataxia, requiring ventilatory support upon discharge.

## Discussion

The association with hydrocephalus is extremely common, occurring in approximately 70%–80% of cases. Dandy-Walker malformations account for 1%–4% of hydrocephalus cases [2]. Leigh syndrome is a severe progressive neurodegenerative disease, manifesting in early childhood, with damage to the nervous system. The most common findings include bilateral and symmetrical focal hyperintensities in several important centers on magnetic resonance imaging. Ultrasonography can be used to diagnose malformations during the prenatal period [3, 4]. However, definitive diagnosis before 18 weeks of gestation is challenging and often only detects classic cases. Treatment of hydrocephalus with shunt significantly reduces mortality rates, but central nervous system defects have a direct correlation with postnatal mortality. The clinical presentation is nonspecific and depends on anatomical alterations; however, it may present with slow psychomotor development, progressive increase in head circumference and tense fontanelle in infants. In preschool children, cognitive deficit is reported in most cases, and signs of cerebellar dysfunction, headache, seizures, and irritability may occur [5, 6].

Treatment revolves around supportive measures for associated problems, with the prognosis varying depending on the associated malformations [7]. The association of variant WD with Leigh’s syndrome, as in the case presented, has not yet been described in the literature, especially due to the difficult diagnosis and possible masking of symptoms due to the overlap of the diseases. Early treatment of neurodegenerative diseases in childhood, with a multidisciplinary team ranging from medical care to intensive treatment with speech therapy and physiotherapy, is necessary for a good prognosis [8]. These measures can prevent or postpone severe dysfunctions, especially hydrocephalus, which is the most common complication, which was avoided in the patient reported.

Hydrocephalus frequently coexists with Dandy-Walker syndrome, occurring in 70%–80% of cases, and the malformation contributes to 1%–4% of all hydrocephalus cases [2]. Leigh syndrome, a severe neurodegenerative disease, typically manifests in early childhood and primarily damages the nervous system. Bilateral and symmetrical lesions are common findings on MRI [3, 4]. Clinicians rely on prenatal ultrasonography to detect malformations, although diagnosing before 18 weeks is challenging and often limited to classic cases. Shunting procedures significantly decrease mortality among hydrocephalus patients, but central nervous system defects correlate strongly with increased postnatal mortality​ [5, 6].

Comprehensive, multidisciplinary treatment (including medical, speech, and physiotherapy support) is essential in managing such complex cases. Early intervention can delay severe dysfunctions, as seen in this patient, and can prevent or lessen the impact of complications like hydrocephalus [7]. The concurrent presentation of Dandy-Walker syndrome with Leigh Syndrome remains rare and challenging to diagnose due to overlapping symptoms. Aggressive and early treatment with a multidisciplinary approach can improve outcomes, helping children with these conditions achieve a better quality of life [8].

## Conclusion

This case underscores the critical importance of early intervention and multidisciplinary care in managing rare congenital and neurodegenerative conditions. With intensive, specialized treatment, it is possible not only to improve quality of life but also to delay the progression of debilitating symptoms. For complex conditions like Dandy-Walker and Leigh syndromes, each advancement in detection and clinical approach offers renewed hope for patients and their families. This study reaffirms that an integrated approach, involving dedicated specialists, can pave the way for promising outcomes in childhood longevity and well-being, offering hope even in the face of the most challenging scenarios.
